# Reviewer acknowledgement 2016

**DOI:** 10.1186/s13039-016-0226-z

**Published:** 2016-02-10

**Authors:** Thomas Liehr, Henry Heng, Yuri Yurov, Aurelia Meloni-Ehrig, Ivan Iourov

**Affiliations:** Jena University Hospital, Institute of Human Genetics, Jena, Germany; Center for Molecular Medicine and Genetics, Wayne State University School of Medicine, Detroit, MI USA; National Research Center of Mental Health, Moscow, Russia; CSI Laboratories, Alpharetta, USA

## Abstract

The Editors of *Molecular Cytogenetics* would like to thank all our reviewers who have contributed to the journal in volume 8 (2015).

**Dilek Aktas**

Turkey

**Mara Almeida**

Brazil

**Daniela Alosi**

Denmark

**Gilda Alves**

Brazil

**Lucilene Arilho Ribeiro-Bicudo**

Brazil

**Rouben Aroutiounian**

Armenia

**István Balogh**

Hungary

**Liming Bao**

USA

**John Barber**

UK

**Iris Bartels**

Germany

**Samarth Bhatt**

USA

**Douglas Bittel**

USA

**Maria Bonaglia**

Italy

**Francesco Brancati**

Italy

**Lukrecija Brecevic**

Croatia

**Helene Bruyere**

Canada

**Joern Bullerdiek**

Germany

**Vincent Cantagrel**

France

**Isabel Maria Marques Carreira**

Portugal

**Chiara Castronovo**

Italy

**Arunrat Chaveerach**

Thailand

**Sau Wai Cheung**

USA

**Richard Choy**

Hong Kong

**Marcelo Cioffi**

Brazil

**Carlos Cordova-Fletes**

Mexico

**Luciana da Silva**

Spain

**Xiangpeng Dai**

USA

**Zunyan Dai**

USA

**Josef Davidsson**

Sweden

**Angelika Dawson**

Canada

**Marc De Braekeleer**

France

**Edivaldo De Oliveira**

Brazil

**Marie Dell' Aquila**

USA

**Jan Diblik**

Czech Republic

**Regen Drouin**

Canada

**Peter Duesberg**

USA

**Andreas Dufke**

Germany

**Emel Ergul**

Turkey

**Carmen Esmer**

Mexico

**Raffaele Falsaperla**

Italy

**Fangpu Fan**

China

**Amanda Figueiredo**

Brazil

**Christopher Franklin**

UK

**Catherine French**

United States

**Radmila Frydrychova**

Czech Republic

**Helen Fryssira**

Greece

**M Fukami**

Japan

**Ioannis Georgiou**

Greece

**Ad Geurts Van Kessel**

Netherlands

**Alexander Graphodatsky**

Russia

**Francesca Grati**

Italy

**Renzo Guerrini**

Italy

**Roberta Guilherme**

Brazil

**Oskar Haas**

Austria

**Claudia Haferlach**

Germany

**Ahmed Hamid**

Denmark

**Karen Harrison**

Canada

**Nyla Heerema**

USA

**Walter Hittelman**

USA

**Ron Hochstenbach**

Netherlands

**Jennelle Hodge**

USA

**Steven Horne**

USA

**Andreas Houben**

Germany

**Galina Hovhannisyan**

Armenia

**Maj Hulten**

UK

**Colleen Jackson-Cook**

USA

**X Ji**

China

**John Kamholz**

USA

**Benjamin Kamien**

Australia

**Elena Kirillova**

Russia

**Genevieve Konopka**

United States

**Nadezda Kosyakova**

Germany

**Dieter Kotzot**

Austria

**Victor Kravetz**

Russia

**Michał Książkiewicz**

Poland

**Anver Kuliev**

USA

**Ingo Kurth**

Germany

**Kwok-Yin Leung**

Hong Kong

**Peining Li**

USA

**Shibo Li**

USA

**Thomas Liehr**

Germany

**Nestor L Lopez Corrales**

Germany

**Isabel López-Expósito**

Spain

**Caroline Mackie Ogilvie**

UK

**Emmanouil Manolakos**

Greece

**Marina Manvelyan**

Armenia

**Ludmila Matyakhina**

USA

**Cristina Mecucci**

Italy

**Aurelia Meloni-Ehrig**

USA

**Martina Merkas**

Croatia

**Patricia Miron**

USA

**Hasmik Mkrtchyan**

Armenia

**Amal Mahmoud Mohamed**

Egypt

**Wagner Molina**

Brazil

**Elisabeth Nacheva**

UK

**Rizwan Naeem**

USA

**Indrajit Nanda**

Germany

**Hiroyuki Nawa**

Japan

**Juergen Neesen**

Austria

**Heike Nelle**

Germany

**Beata Nowakowska**

Belgium

**Moneeb Othman**

Germany

**Hong Pan**

China

**Davide Pareyson**

Italy

**Francesco Pasquali**

Italy

**Lorenza Pecciarini**

Italy

**Michael B. Petersen**

Greece

**Petr Ráb**

Czech Republic

**Maryam Rafati**

Iran

**Martina Rincic**

Croatia

**Anna Rita Rossi**

Italy

**Elena Rossi**

Italy

**Feride Sahin**

Turkey

**Heather Sanders**

USA

**Damien Sanlaville**

France

**Heide Schatten**

USA

**Albert Schinzel**

Switzerland

**Brigitte Schlegelberger**

Germany

**Samantha Schrier Vergano**

USA

**Marco Seri**

Italy

**Harsh Sheth**

UK

**Frenny Sheth**

India

**Malte Spielmann**

Germany

**Jeremy Squire**

Brazil

**Pawel Stankiewicz**

USA

**Zornitza Stark**

Australia

**Maria Syrrou**

Greece

**Minghan Tong**

China

**Vladimir Trifonov**

Russia

**Angelo Valetto**

Italy

**Joris Vermeesch**

Belgium

**Sonja Vernes**

Netherlands

**E. M. Vestergaard**

Denmark

**Victoria Voinova**

Russia

**Emanuela Volpi**

UK

**Svetlana Vorsanova**

Russia

**Xinjing Wang**

USA

**Dorothy Warburton**

USA

**Jingly Weier**

USA

**Anja Weise**

Germany

**Kathleen Wilhelm**

Germany

**Dan Xu**

USA

**Zhihong Yang**

USA

**Cassia Yano**

Brazil

**Adnan Younis**

South Korea

